# Non-Alcoholic Fatty Liver Disease in Overweight Children: Role of Fructose Intake and Dietary Pattern

**DOI:** 10.3390/nu10091329

**Published:** 2018-09-19

**Authors:** Anika Nier, Annette Brandt, Ina Barbara Conzelmann, Yelda Özel, Ina Bergheim

**Affiliations:** 1 Department of Nutritional Sciences, Molecular Nutritional Science, University of Vienna, A-1090 Vienna, Austria; anika.nier@univie.ac.at (A.N.); annette.brandt@univie.ac.at (A.B.); 2 Department of Nutritional Medicine, (180), University of Hohenheim, D-70599 Stuttgart, Germany; ina.conzelmann@gmx.de (I.B.C.); yelda@directbox.com (Y.Ö.)

**Keywords:** children, overweight, NAFLD, fructose, dietary pattern, dietary intervention

## Abstract

The role of nutrition and diet in the development of non-alcoholic fatty liver disease (NAFLD) is still not fully understood. In the present study, we determined if dietary pattern and markers of intestinal permeability differ between overweight children with and without NAFLD. In addition, in a feasibility study, we assessed the effect of a moderate dietary intervention only focusing on nutrients identified to differ between groups on markers of intestinal barrier function and health status. Anthropometric data, dietary intake, metabolic parameters, and markers of inflammation, as well as of intestinal permeability, were assessed in overweight children (*n* = 89, aged 5–9) and normal-weight healthy controls (*n* = 36, aged 5–9). Sixteen children suffered from early signs of NAFLD, e.g., steatosis grade 1 as determined by ultrasound. Twelve children showing early signs of NAFLD were enrolled in the intervention study (*n* = 6 intervention, *n* = 6 control). Body mass index (BMI), BMI standard deviation score (BMI-SDS), and waist circumference were significantly higher in NAFLD children than in overweight children without NAFLD. Levels of bacterial endotoxin, lipopolysaccharide-binding protein (LBP), and proinflammatory markers like interleukin 6 (IL-6) and tumor necrosis factor α (TNFα) were also significantly higher in overweight children with NAFLD compared to those without. Total energy and carbohydrate intake were higher in NAFLD children than in those without. The higher carbohydrate intake mainly resulted from a higher total fructose and glucose intake derived from a significantly higher consumption of sugar-sweetened beverages. When counseling children with NAFLD regarding fructose intake (four times, 30–60 min within 1 year; one one-on-one counseling and three group counselings), neither alanine aminotransferase (ALT) nor aspartate aminotransferase (AST) activity in serum changed; however, diastolic blood pressure (*p* < 0.05) and bacterial endotoxin levels (*p* = 0.06) decreased markedly in the intervention group after one year. Similar changes were not found in uncounseled children. Our results suggest that a sugar-rich diet might contribute to the development of early stages of NAFLD in overweight children, and that moderate dietary counseling might improve the metabolic status of overweight children with NAFLD.

## 1. Introduction

Non-alcoholic fatty liver disease (NAFLD) comprises a wide spectrum of diseases ranging from simple steatosis afflicted with fat accumulation to steatohepatitis, fibrosis, and even cirrhosis or hepatocellular carcinoma [1]. Results of epidemiological studies suggest that NAFLD is, by now, the most prevalent liver disease in the world [2]. Contrary to many other liver diseases, NAFLD is not a disease only found in adults, but, with a still increasing prevalence, is estimated to also affect 8% of normal-weight and up to 34% of overweight children and adolescents [3,4,5]. Despite intense research efforts, mechanisms underlying the onset and progression of the disease are still not fully understood, and therapies other than lifestyle interventions are not yet available. However, lifestyle interventions, be it general caloric restriction diets, or low-fat or low-carbohydrate diets with or without increased physical activity, are frequently afflicted with low compliance and high drop-out, as well as relapse rates, in both children and adults [6,7,8].

Similar to the results in epidemiological and clinical studies [9,10,11,12], results of animal studies analyzing the role of diet in the development of NAFLD, as well as mechanisms involved, suggest that both general overnutrition and a diet rich in certain macronutrients like saturated fat and/or sugars like fructose are critical in the onset of NAFLD [13,14,15,16]. For example, it was shown that not only obese (ob/ob) mice, but also mice fed a diet rich in saturated fats and/or fructose develop liver steatosis and early signs of hepatic inflammation within several weeks [17,18,19]. Furthermore, it was repeatedly shown in these dietary models that the development of NAFLD was associated with alterations of intestinal barrier function and elevated bacterial endotoxin levels. In line with these findings, results of our own group and others suggest that, in adults and children with NAFLD, the development of the disease is not only associated with general overnutriton, but also, frequently, an elevated intake of fructose and fat, as well as alterations of markers of intestinal barrier function [10,12,20,21,22]. However, whether or not general overnutriton or the intake of a specific dietary pattern is critical in the development of NAFLD and associated intestinal barrier dysfunction is yet to be fully clarified.

Starting from this background, the aim of the present study was to determine if the dietary pattern and lifestyle of overweight children without NAFLD differs from overweight children showing early signs of NAFLD. Accordingly, overweight children randomly recruited in primary schools, sports clubs, and kindergartens were stratified by results of liver ultrasounds in overweight children with and without NAFLD. For comparison, a group of normal-weight healthy children were enrolled in the study. Furthermore, to determine if moderate dietary counseling, focusing only on parameters identified to be critical in the dietary pattern of overweight children with NAFLD, is an approach to improve the health status of overweight children with NAFLD, some of the overweight children with NAFLD were enrolled in a feasibility study.

## 2. Materials and Methods

### 2.1. Subjects

All children were recruited from the so-called “Hohenheim Fructose Intervention (HoFI) study” registered at http://clinicaltrials.gov (NCT01306396). The ethics committee of the Landesärztekammer Baden-Württemberg (Stuttgart, Germany) approved the present study, which was then performed in accordance with the ethical standards laid down in the Declaration of Helsinki (2008). Participants were recruited between April 2009 and December 2010 primarily through elementary schools in Stuttgart and the greater area of Stuttgart, Southern Germany. All subjects and their guardians gave written informed consent to participate in the study. A total of 92 overweight children aged 5–9 years without any known signs of NAFLD or other metabolic diseases before being enrolled in the study were included, while 3 children had to be excluded from the baseline analysis due to underreporting (overweight children without NAFLD: *n* = 2; overweight children with NAFLD: *n* = 1). Of the 17 overweight children identified to suffer from early stages of NAFLD, all agreed to participate in the feasibility study detailed below. Eight of the overweight children with NAFLD were selected to be in the intervention group and nine agreed to serve as controls. Despite selecting the groups, four of the 17 children enrolled in the intervention study dropped out of the study for personal reasons (three controls and one intervention child), and one child had to be excluded from the final analysis of the feasibility study as it was identified as a case of underreporting (intervention group). In addition, data from 36 healthy normal-weight children aged 5–9 reported in previously published studies [21,23] were included in the study for comparison. None of the children included had a known history of (i) steatohepatitis, (ii) renal insufficiency, (iii) diabetes type 1 and 2, (iv) chronic diseases of the gastrointestinal tract, or (v) taking lipid-lowering drugs or drugs affecting lipid metabolism. All children enrolled in the study were prepubertal as defined by Marshall and Tanner [24,25]. Pubertal status was assessed as described by Maier et al. [26]. None of the children enrolled in the feasibility study changed pubertal status throughout the study.

### 2.2. Laboratory Measurements, Blood Pressure, and Abdominal Ultrasound

From all participants, the following parameters were assessed in a routine laboratory (Sindelfingen, Germany) as described previously [27]: alanine aminotransferase (ALT), aspartate aminotransferase (AST), fasting blood glucose, blood lipids, and uric acid in serum, as previously described in a fasting venous blood sample. In addition, concentrations of active plasminogen activator inhibitor 1 (PAI-1; LOXO, Dossenheim, Germany), lipopolysaccharide-binding protein (LBP; Abnova, Taipei City, Taiwan), leptin and insulin (both Hölzel GmbH, Wildberg, Germany), tumor necrosis factor α (TNFα; IBL international GmbH, Hamburg, Germany), adiponectin (TECOmedical AG, Sissach, Switzerland), c-reactive protein (CRP; DRG Instruments GmbH, Marburg, Germany), and interleukin 6 (IL-6; R&D Systems, Abingdon, UK) were determined in plasma using commercially available ELISA kits. Systolic and diastolic blood pressure, as well as liver status using ultrasound, were measured and defined as detailed previously [27].

### 2.3. Bacterial Endotoxin

Plasma endotoxin levels were determined by an endpoint enzymatic assay based on limulus amebocyte lysate, as detailed previously [12]. 

### 2.4. Glucose Metabolism

Glucose tolerance was determined as detailed previously [26]. The homeostasis model assessment for insulin resistance (HOMA-IR) index (HOMA-IR = (fasting insulin (µIU/mL) × fasting glucose (mmol/L))/22.5) was used to determine insulin resistance.

### 2.5. Assessment of Dietary Intake and Leisure Time Activities

Dietary intake using two separated 24-h recalls, including one weekend day, as well as sportive and sitting leisure time activities, was assessed as previously described [28]. For analyzing nutritional intake, the EBISpro software was used. This software contains the German Nutrient Database (in German: Bundeslebensmittelschlüssel, see also https://www.blsdb.de) which includes data of about 10,000 foods and covers average nutritional values, including free fructose (137 constituent data per food item). Dietary underreporting was determined by calculating the ratio of reported total energy intake and predicted individual basal metabolic rate, as described previously by others [29]. Only recalls with ratios above the age- and sex-specific cut-off values being 1.04 for boys and 1.01 for girls were included in the nutritional analysis [30]. Based on this calculation, three underreporters were identified (overweight children without NAFLD: *n* = 2, overweight children with NAFLD: *n* = 1) and excluded from the final analysis and the analysis of the feasibility study, respectively.

### 2.6. Anthropometric Measurements and Socio-Demographic Data

Anthropometric parameters were assessed by a nutritionist as detailed by Maier et al. [26]. Furthermore, socio-demographic characteristics such as age, gender, and ethnicity were recorded.

### 2.7. Dietary Intervention Study

**Figure 1:**
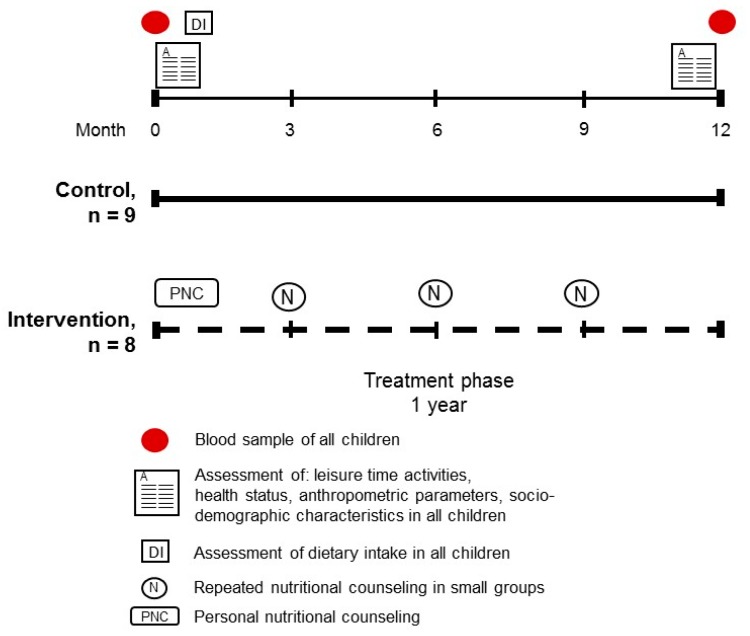
Study design of the feasibility study assessing the effect of a moderate dietary intervention focusing only on the reduction of fructose intake (−50%) on the health status of overweight children with non-alcoholic fatty liver disease (NAFLD).

In children enrolled in the feasibility study, all measurements assessing health status and anthropometry were repeated after one year. As the feasibility study required a high compliance of participants in the intervention group, e.g., attending regular meetings (one one-on-one meeting and three group meetings), and altering dietary patterns and intake throughout the year of study, study participants and their guardians were allowed to choose study arms to avoid further drop-out (for study design, see also Figure 1). Based on daily fructose intake as determined in two independently performed 24-h recalls before the study, children enrolled in the intervention group were advised to reduce their daily fructose intake by ~50% in a personal nutritional counseling taking place at the beginning of the study in the presence of their respective guardians. Counseling was then repeated every three months in small groups (see Figure 1). Children, as well as guardians, were counseled independently. Training of both children and guardians focused primarily on a change in dietary pattern and included strategies to identify fructose-rich foods, e.g., foods containing marked amounts of sucrose or fructose, such as lemonades, chocolates, cookies, cakes, and candies, as well as foods containing large amounts of free fructose, such as juices, certain fruits, and vegetables, and to replace these foods with foods containing less fructose of the same food category (e.g., exchanging cookies with rusk, replacing sucrose with glucose when making cakes or cookies, or to consume diet lemonades instead of sugar lemonades (for details, see Reference [27])). In brief, before the study, several games, quizzes, and experiments suitable for training children were developed and their feasibility was assessed in a pilot study [27]. For example, to illustrate the effect of sugar-sweetened beverages on dental health, a tooth was put into a glass containing a sugar-sweetened beverage for two days. Furthermore, children were asked to guess the number of sugar cubes contained in different foods (e.g., ketchup, chocolate bars, and sugar-sweetened beverages) by selecting glasses filled with different numbers of sugar cubes to enhance the awareness of sugar content in commercially available products. Additionally, a small supermarket with food dummies was built to practice children’s behavior in the supermarket. Children were then asked to pick their favorite foods, and, together with a trained nutritionist, foods with high amounts of sugars were identified and alternative foods with lower sugar content were selected. Upon request, participants and their respective guardians enrolled in the control group received one dietary counseling based on general recommendations for a healthy nutrition as recommended by the German Nutrition Society.

### 2.8. Statistical Analyses

Data were analyzed using the *t*-test and Mann–Whitney U test for comparing overweight children with and without NAFLD and to compare baseline and end values of children enrolled in the pilot feasibility study. Healthy, normal-weight children were not included in the statistical analysis, but are shown for comparison. The Wilcoxon test was used to compare baseline and end values within the two groups enrolled in the feasibility study. Fisher’s exact test was used for comparing gender and ethnicity (GraphPad Prism, version 7.03, 2017, GraphPad Software Inc., San Diego, CA, USA). Identified outliers using Grubbs’ test were excluded from baseline analyses. As mentioned above, children identified as underreporters were removed from the final analyses and the analysis of the feasibility study. A *p*-value ≤0.05 was defined as the level of significance.

## 3. Results

### 3.1. Characteristics of the Study Participants

**Figure 2:**
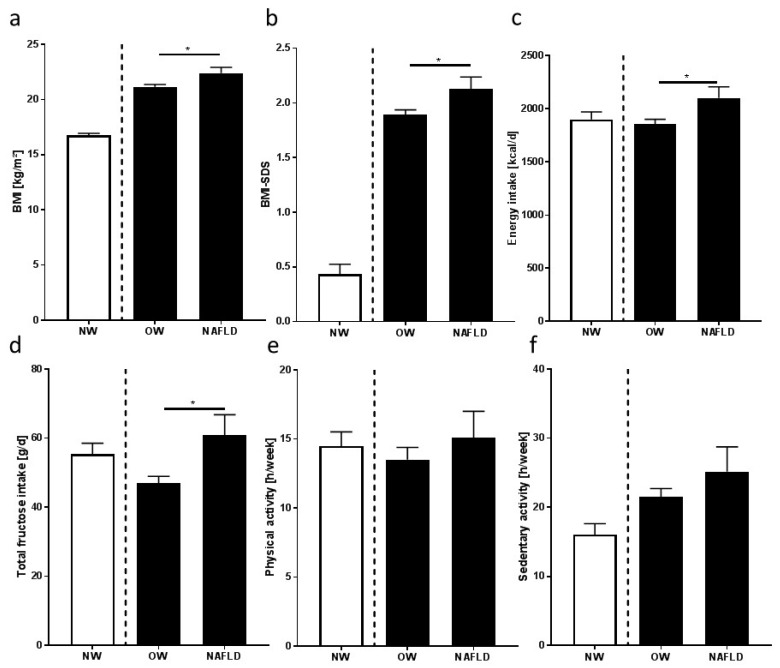
(**a**) Body mass index (BMI), (**b**) BMI standard deviation score (BMI-SDS), (**c**) energy, (**d**) total fructose intake (free fructose and fructose derived from sucrose), and (**e**) physical and (**f**) sedentary activities of normal-weight (NW) children, overweight children without NAFLD (OW), and overweight children with NAFLD (NAFLD). Data are means ± standard error of the mean (SEM), * *p* < 0.05 overweight children in comparison to overweight children with NAFLD; NW children were not included in the statistical analysis, but are shown for comparison. Underreporters were excluded from the analysis.

As shown in Table 1, neither age nor distribution of gender or ethnicity differed between overweight children with and without early signs of NAFLD. Body weight was significantly higher in overweight children with NAFLD than in those without, while height was similar. Accordingly, body mass index (BMI), BMI standard deviation score (BMI-SDS), and waist circumference were also significantly higher in overweight children with NAFLD (see Table 1 and Figure 2). Neither leptin nor adiponectin, nor ratio of leptin and adiponectin differed between overweight groups. Still, as expected, all of these parameters differed markedly from those of normal-weight children who were included in the study for comparison only (see Table 1).

Despite showing early signs of NAFLD, e.g., fatty liver grade 1, ALT activity in serum was similar between overweight children with and without NAFLD, while AST activity in serum was significantly higher by ~4 U/L (*p* < 0.05). Systolic and diastolic blood pressures were similar between groups of overweight children. Twelve of the overweight children without NAFLD and seven of the overweight children with NAFLD were diagnosed as suffering from hypertension. Triglyceride, high-density lipoprotein (HDL), and uric acid concentrations in serum were also similar between the two overweight groups with 21 overweight children and four overweight children with NAFLD suffering from dyslipidemia. However, low-density lipoprotein (LDL) and total cholesterol were significantly higher in overweight children without NAFLD than in overweight children with NAFLD. Fasting insulin, glucose, and HOMA-IR were also similar between overweight groups. Furthermore, three overweight children and one overweight child with NAFLD suffered from an impaired glucose tolerance according to the reference levels of the German diabetes association [31]. As expected and similar to anthropometric parameters, metabolic parameters were markedly higher, or, in the case of HDL, lower in overweight children with and without NAFLD than in normal-weight children shown for comparison (see Table 1).

### 3.2. Nutritional Intake, Dietary Pattern, and Leisure Time Activities

In line with the findings for body weight, BMI, and BMI-SDS, total caloric intake of overweight children with NAFLD was significantly higher than that of overweight children without NAFLD (~250 kcal/day). Total fat, protein, and fiber intakes were similar between overweight groups (see Table 2 and Figure 2). Total intake of carbohydrates was by trend higher in overweight children with NAFLD than in those without (~120 kcal/day, *p* = 0.06). As results of others and our own group suggest that intake of carbohydrates and, herein, especially mono- and disaccharides may be critical in the development of NAFLD [10,32,33], the composition of the carbohydrates was further analyzed. While the intake of complex carbohydrates was similar between overweight groups, intakes of total fructose and total glucose (free fructose and glucose, respectively, as well as fructose and glucose derived from sucrose) were significantly higher in overweight children with NAFLD than in those without (see Table 2 and Figure 2).

To further determine dietary sources of higher carbohydrate, and especially, monosaccharide intake, we analyzed the dietary pattern of study participants (see Table 3). With the exception of cereals being consumed by ~47% of overweight children without NAFLD and only ~13% of overweight children with NAFLD (*p* < 0.05), the intake of food groups was similar between overweight groups (data not shown). However, when further analyzing the amount consumed of the different food groups of those children reporting consumption of these foods, overweight children with NAFLD were found to consume significantly more sweetened beverages including soft drinks and fruit juices than overweight children without NAFLD. Intake of all other food groups was similar between groups (see Table 3).

Times spent with physical and sedentary activities were similar between overweight groups. Interestingly, the intake of total calories and macronutrients, as well as time spent with physical activities, of normal-weight children was rather similar to that of overweight children with NAFLD, while time spent with sedentary activities was markedly shorter in normal-weight children than in overweight ones (see Table 1 and Figure 2). Again, underreporters were excluded from the analysis.

### 3.3. Markers of Inflammation and Intestinal Permeability

**Figure 3:**
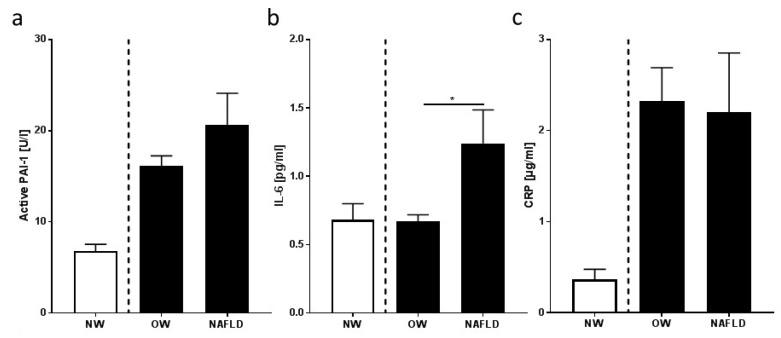

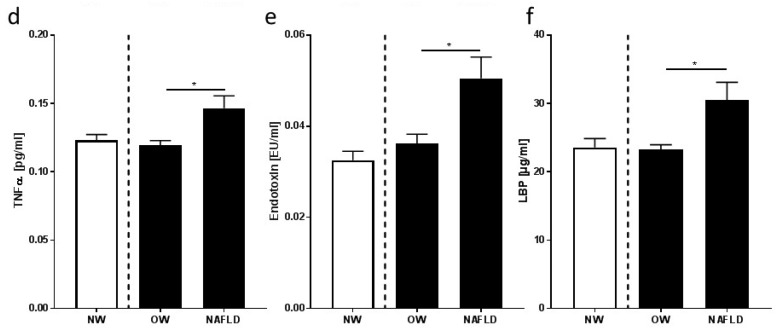
(**a**) Plasma active plasminogen activator inhibitor 1 (PAI-1), (**b**) interleukin 6 (IL-6), (**c**) serum c-reactive protein (CRP), (**d**) tumor necrosis factor α (TNFα), (**e**) endotoxin, (**f**) lipopolysaccharide-binding protein (LBP) plasma concentrations of normal-weight (NW) children, overweight children without NAFLD (OW), and overweight children with NAFLD (NAFLD). Data are means ± SEM; * *p* < 0.05 overweight children in comparison to overweight children with NAFLD; NW children were not included in the statistical analysis, but are shown for comparison. Underreporters were excluded from the analysis.

Protein concentrations of TNFα and IL-6 were significantly higher in plasma of overweight children with NAFLD than in those without, while concentrations of CRP and active PAI-1 in plasma were similar between overweight groups (see Figure 3). Bacterial endotoxin levels in peripheral plasma and protein levels of LBP were both significantly higher in overweight children with NAFLD than in those without (see Figure 3).

### 3.4. Feasibility Study: Characteristics of Study Participants, Nutritional Intake, and Metabolic Parameters

**Figure 4:**
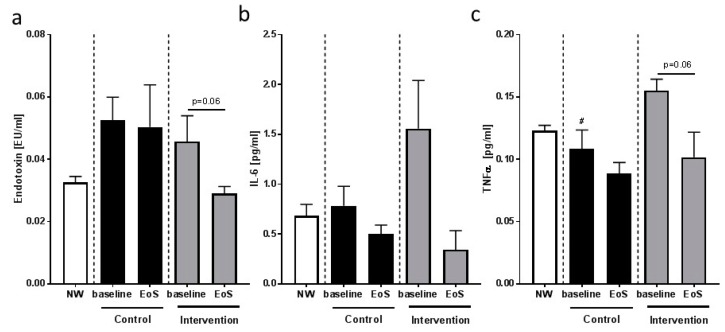
(**a**) Plasma endotoxin, (**b**) serum IL-6, and (**c**) plasma TNFα concentrations of children with NAFLD enrolled in the control (Control) and intervention (Intervention) group at baseline (baseline) and at the end of the study (EoS). Data are means ± SEM; ^#^ *p* < 0.05 values at baseline between both groups; NW children were not included in the statistical analysis, but are shown for comparison. Underreporters were excluded from the analysis.

Four children dropped out of the feasibility study due to personal reasons, and one child was excluded as an underreporter. Therefore, only six children in the control group and six children in the intervention group were included in the final analysis. Despite being allowed to select study arms to enhance compliance and to avoid the loss of children, at baseline, none of the parameters assessed differed between the overweight children with NAFLD selecting the intervention arm and those who chose to participate in the feasibility study as controls, with the exception of TNFα levels in plasma (see Figure 4). TNFα levels in plasma were significantly higher in children in the intervention group than in controls. Additionally, total intakes of energy, macronutrients, and sugars (total fructose and total glucose) per day were similar between groups (see Supplementary Materials, Table S1) at baseline. Data of normal-weight children in Table 4 and Figure 4 are shown for comparison, but were not included in the statistical analysis. While both groups had lower BMI-SDS at the end of the study compared to the beginning of the study (control: *p* < 0.05; intervention: *p* = 0.16), waist circumference was significantly higher in the intervention group at the end of the study (see Table 4). A similar increase in waist circumference was not found in the controls. Nonetheless, waist circumference did not differ between groups at the end of the study. In line with the findings for waist circumference, neither adiponectin nor leptin plasma concentrations nor leptin/adiponectin ratio differed at baseline. While leptin levels remained unchanged, adiponectin concentrations in the plasma of children in the intervention group were by trend lower (*p* = 0.06) at the end of the study when compared to baseline, whereas leptin/adiponectin ratio was significantly higher in these children.

ALT and AST activities, as well as concentrations of triglycerides and HDL in serum, were not altered throughout the intervention study and did not differ between NAFLD groups. Fasting insulin and glucose concentrations, as well as HOMA-IR, were also similar between groups at the beginning of the study and were not changed throughout the study (see Table 4). However, LDL and total cholesterol levels increased significantly throughout the study in the sera of children in the intervention group. LDL concentrations in serum also increased in the control group; however, as data varied considerably within these groups, differences did not reach statistical significance. The systolic blood pressure of children was similar between groups at the beginning of the study and was also not changed. In contrast, diastolic blood pressure being similar between groups at baseline was significantly lower in children in the intervention group at the end of the study, whereas a similar decrease was not found in control NAFLD children (see Table 4).

### 3.5. Feasibility Study: Inflammatory Markers and Indices of Intestinal Permeability

Concentrations of active PAI-1 and IL-6 were similar between groups at the beginning of the study and were not altered. While changes in concentration of these inflammatory markers were not significant within groups or between groups, at the end of the feasibility study, concentrations of most parameters were at the level of normal-weight children (see Table 4 and Figure 4). In contrast, plasma TNFα levels, being significantly higher in the intervention group at baseline compared to controls, decreased markedly (*p* = 0.06) throughout the study almost to the level of normal-weight control children (see Figure 4). Bacterial endotoxin and LBP concentrations in plasma were also similar between groups at the beginning of the study. LBP concentration in plasma was unchanged in both groups at the end of the study (see Table 4), whereas the concentration of bacterial endotoxin in the peripheral blood of children in the intervention group was lower by trend at the end of the study (*p* = 0.06; see Figure 4).

## 4. Discussion

With a still increasing prevalence, NAFLD is, by now, thought to be the most prevalent liver disease worldwide [2]. Overweight and insulin resistance are among the key risk factors for the development of NAFLD [34,35]; however, despite intense research efforts, the question as to why some overweight individuals develop NAFLD and others do not is yet to be fully answered. In the present study, employing a cohort of randomly selected overweight children with no known signs of liver disease or other metabolic diseases before the study, it was shown that children with early signs of NAFLD, e.g., fatty liver grade 1 as assessed by ultrasound, had a higher BMI, BMI-SDS, and waist circumference than overweight children without NAFLD. Also, while still being within the normal range, AST activity was higher in the sera of overweight children with NAFLD, whereas neither ALT activity nor markers of glucose metabolism nor other metabolic markers, such as blood pressure and triglycerides, differed between groups. Somewhat contrasting the findings for waist circumference, the ratio of adiponectin to leptin, suggested to be indicative of visceral fat volume and NAFLD in obese adolescents [36], was similar between overweight groups. However, results of a recently published study by Dhaliwal et al. [37] using computed tomography to assess hepatic steatosis and the abdominal fat area suggest that, while abdominal subcutaneous adipose tissue per se is greater in children with hepatic steatosis than in those without, increases in visceral adipose tissue area seem to be related to the presence of steatosis in older children (≥9.8 years). Therefore, the apparent lack of relation of waist circumference and the ratio of adiponectin to leptin might be related to the rather young age of study participants (<9 years) and to differences in subcutaneous adipose tissue mass rather than visceral adipose tissue. Furthermore, results of others also suggested that overweight children with hepatic steatosis have a higher BMI and waist circumference when compared to overweight children without signs of NAFLD [5,38,39]. Previous studies of others also reported that transaminase activities in the sera of overweight children were also similar to those of overweight children serving as controls [38]; however, in contrast to the findings of the present study, in studies of others, a strong association of the presence of NAFLD in overweight children with increased markers of insulin resistance, dyslipidemia, and the presence of metabolic abnormalities was found [5,38,39]. Indeed, in the present study, total cholesterol in serum was even found to be higher in overweight children without NAFLD than in those with NAFLD. Differences between the results of these studies (>9 years old) and the present study (<9 years old) might have resulted from differences in the age of study participants, as well as in the severity of steatosis (in the present study, only grade I vs. minimal-to-severe fatty liver or even beginning non-alcoholic steatohepatitis (NASH) in other studies) and obesity of participants enrolled [5,38]. The study setting (here, children recruited in schools vs. hospitals in most other studies) and markers used to assess insulin resistance (in the present study, fasting glucose and insulin vs. oral glucose tolerance tests) were also markedly different between these studies and the present study [5,38,39]. Reasons for the significantly higher levels of total cholesterol and LDL cholesterol in the sera of overweight children without NAFLD have to be delineated in future studies.

Also, while overweight children with NAFLD only showed very early signs of the disease, IL-6 and TNFα levels in plasma were both higher in children with NAFLD than in overweight controls, whereas CRP and active PAI-1 levels in plasma—both markedly higher than in normal-weight controls—did not differ between overweight groups. In line with these findings, others showed that concentrations of IL-6 and TNFα in serum are closely related to the prevalence of NALFD [40]. Results of others also suggest that, in overweight children, CRP and active PAI-1 levels may be elevated independently of the presence of NAFLD [41,42]. Indeed, while active PAI-1 levels were shown to be related to severe stages of the disease, e.g., manifest steatosis or NASH [43], this acute-phase protein was also shown to be related to HOMA-IR by others [44], and it was similarly higher in both overweight groups in the present study when compared to controls. Taken together, results of the present study suggest that, in overweight children, very early stages of NAFLD are associated with higher body weight, greater waist circumference, and elevated proinflammatory cytokine levels while, markers of insulin resistance are not different. However, the results of the present study by no means preclude that an impaired glucose tolerance or insulin resistance contributes to the onset of NAFLD. Indeed, in adults and mouse models, it was shown that both fasting insulin and glucose levels can still be within the normal range in peripheral blood, while, in liver tissue, the expressions of insulin receptor and insulin receptor substrate were markedly lower [45,46]. Therefore, it could be that, in the present study, overweight children with NAFLD may have suffered from impairments of insulin signaling and glucose metabolism in liver tissue, while fasting glucose and insulin concentrations in peripheral blood were still within the normal range. This needs to be addressed in future studies.

### 4.1. Absolute Energy Intake, Nutritional Intake, and Dietary Pattern of Overweight Children with and without NAFLD Differ

Results of animal studies suggest that not only general overnutrition, but also the composition of the diet, e.g., the proportion of saturated fatty acids and sugars, and herein, especially of fructose, may be critical in the development of NAFLD [19,47]. In a cohort of children with NAFLD, Mosca et al. [33] recently showed that dietary fructose intake is independently associated with NASH. Furthermore, it was shown that children with NAFLD absorb and metabolize fructose more effectively than normal-weight children [48]. In the present study, overweight children with early signs of NAFLD had a significantly higher mean daily total energy intake when compared to overweight children without NAFLD (~250 kcal/day) which mainly seemed to result from a higher daily total fructose (free fructose and fructose derived from sucrose) and total glucose (free glucose and glucose derived from sucrose) intake originating from a markedly higher soft-drink and juice intake. Results of the present study are in line with the findings of others, showing that both children and adults with NAFLD have a higher mean fructose intake mainly resulting from a higher consumption of soft drinks and fruit juices [10,49,50,51]; however, in most of these studies, normal-weight healthy individuals were compared with overweight patients with NAFLD [10,12]. Indeed, the number of human studies comparing the nutritional intake and dietary pattern of overweight individuals, and even more so, weight-matched individuals with and without NAFLD is rather limited. In line with the findings of the present study, Ouyang et al. and Assy et al. [52,53] showed that adult patients with NAFLD drank more soft drinks and juices.

Interestingly, normal-weight children enrolled for comparison almost had similar daily total energy, monosaccharide, and disaccharide intakes without showing any signs of NAFLD or other metabolic diseases when compared to overweight children with NAFLD. Yet, normal weight children, on average, were ~16 h/week (~140 min/day) sedentarily active doing handcrafting, drawing, reading, and watching TV or playing video games, while overweight children with NAFLD, on average, spent 25 h/week (~215 min/day) with these activities. Indeed, results of Felix et al. [54] suggest that, in overweight children, the intake of refined carbohydrates and the lack of physical activity were associated with a higher risk of developing NAFLD, further suggesting that protection against the development of NAFLD, and probably, overweight in normal-weight children was strongly dependent upon their physical activity in the present study. In support of this hypothesis, lifestyle changes not only focusing on changes in dietary habits, but also on increasing aerobic exercise were suggested to improve aminotransferase activity levels in children and adolescents with NAFLD [50,55].

### 4.2. Overweight Children with NAFLD Have Higher Bacterial Endotoxin and LBP Levels in Peripheral Blood Than Overweight Children without NAFLD, Which Were Lowered by Moderate Dietary Counseling

In the present study, both bacterial endotoxin and LBP levels were significantly higher in overweight children with NAFLD than in those without. These findings are in line with the results of several human and animal studies, suggesting that alterations of intestinal barrier function, and subsequently, an increased translocation of bacterial endotoxin are critical in the development of NAFLD [10,12,17,21,56,57,58]. While data derived from animal studies suggest that these alterations may be related to the intake of fructose [14,19,58], results of human studies are somewhat contradictory. Indeed, in some studies, it was shown that both the intake of dietary fructose and bacterial endotoxin levels in peripheral blood were elevated in patients with NAFLD; however, frequently, not only fructose intake, but also total caloric intake of patients was significantly higher than that of controls [49,53]. So far, studies focusing on a reduction of fructose intake suggest that, in adults with steatosis and steatohepatitis, this kind of counseling is associated with an improvement in liver status, a reduction in bacterial endotoxin levels, and improved intestinal barrier function [59]. In the present feasibility study, while only slightly affecting overall weight and metabolic status, the moderate dietary counseling focusing only on a reduction in dietary fructose intake was associated with a reduction in bacterial endotoxin and TNFα levels, almost to the level of normal-weight controls, in overweight children with NAFLD. The apparent reduction in adiponectin levels from baseline to the end of study in children with NAFLD enrolled in the intervention arm might have resulted from the slightly, but not significantly, younger age of these children when compared to controls at baseline (7.5 vs. 8.0 years). Indeed, it was shown before that adiponectin plasma levels decrease between the ages of five and eight years [60]. Furthermore, studies of Murphy et al. [60] also showed that total cholesterol plasma levels increase over time in children aged between five and eight years, in line with findings of the present study. In contrast, despite lowering their BMI-SDS and maintaining their waist circumference, the bacterial endotoxin levels of overweight controls with NAFLD were unchanged. Taken together, these data suggest that the total intake of sugar-rich foods and bacterial endotoxin levels both may be critical in the development of NAFLD in overweight children. However, our results do not preclude that other factors such as genetic predisposition, intake of other nutrients, and sedentary lifestyle are also critical in the development of NAFLD. Rather, our data suggest that, at least in some children, targeting intestinal barrier function through dietary fructose intake may be beneficial in the prevention and therapy of this liver disease.

### 4.3. Limitations

Our study is not without limitations which have to be considered when interpreting the results. Overweight children with and without NAFLD were not weight-matched; however, children were randomly recruited and enrolled in non-clinical settings, and, at the time of recruitment, had no known history of metabolic or liver diseases. Therefore, both overweight groups included metabolically healthy and unhealthy children. Thus, results might differ in larger and more homogeneous clinical studies. Nonetheless, as we aimed to study the early onset of the disease, this approach seemed to be the most feasible. Furthermore, the sample size of the intervention study was rather small, as the intervention focused only on children with NAFLD and five children were lost due to personal reasons or underreporting. Furthermore, as the focus of the intervention study was to show feasibility of this kind of moderate dietary intervention, no power calculation was performed to determine the number of subjects needed to be included for statistically significant outcomes. Thus, the characteristics of the feasibility study are rather explorative, and the effect of a moderate dietary intervention on metabolic and inflammatory markers needs to be assured in a larger randomized population. However, despite the small sample size, our findings are in line with others showing that dietary counseling might be beneficial in improving metabolic parameters in children [61]. Furthermore, a selection bias cannot be ruled out as control and intervention groups were self-selected due to incompliance of many guardians for randomization. Indeed, due to drop out and underreporting, the number of Asian participants deciding to undergo nutritional counseling was lower than the number of Asian children in the control group. However, as we were highly dependent upon the willingness of parents and children to attend the regular counseling meetings, from our perspective, this was the most feasible way of avoiding an even higher drop-out rate. Indeed, when enrolling children into the study, guardians repeatedly pointed out their unwillingness to participate in regular meetings, suggesting that it felt too straining. Accordingly, it was also not possible to obtain valid data regarding nutritional intake and dietary pattern at the end of the intervention. Therefore, it is not clear if the beneficial effects on bacterial endotoxin levels found at the end of the study resulted from a change in fructose intake or dietary pattern, or other factors. The role and impact of fructose on the beneficial effects found in the intervention group will have to be addressed in future studies. Reasons for this incompliance to participate in meetings might have been that our study was not situated in a clinical setting, and that children were thought to be healthy with the exception of being overweight/ obese before the study. Another limitation is the reduction of BMI-SDS in both groups of the intervention study. This, in part, might have resulted from the fact that guardians became aware of the potential health issues of their children. Indeed, some of the families might have changed additional dietary habits, and prolonged time spent physically active, while time spent sedentary active was reduced, without bringing this to our attention. Furthermore, in the region of Germany where our study was situated, “healthy” nutrition is part of the curriculum in elementary school and sometimes even in kindergarten. Therefore, it cannot be ruled out that at least some of the children in the intervention study and probably also their parents, e.g., during parent–teacher conferences and school enrollment, might have received additional training in regards to avoiding sugar-rich foods and to following a healthy lifestyle. Furthermore, in the present study, physical and sedentary activities were only acquired by questionnaires rather than activity monitors. Additionally, no follow-up was carried out to assess sustainability of the intervention on weight status, metabolic disorders, and associated proinflammatory alterations. However, we thought that the length of the study would be sufficient to test the principal hypothesis that, in children, a diet focusing only on a reduction in fructose intake may be a sufficient measure for reducing bacterial endotoxin levels and concentrations of proinflammatory cytokines. Long-term effects will have to be determined in larger randomized studies with a longer duration and follow-up.

## 5. Conclusions

Taken together, results of the present study suggest that body weight, dietary pattern, and especially, the intake of sweetened beverages may be critical in the development of NAFLD in overweight children. Our data also suggest that changes in intestinal barrier function are also associated with the development of NAFLD in children. Results of the present study also suggest that targeting sugar or fructose intake even with moderate measures may be beneficial for overall health status of overweight children with NAFLD. Still, the concept of moderate lifestyle interventions only focusing on a limited number of changes and the long-term effects of interventions like the one used in the present study need to be assessed in larger randomized studies in the future, as, in the present study, the sample size was quite small and participants were allowed to self-select their groups. Also, future studies should maybe aim to include more family members in the intervention, so as to improve the motivation to participate in and attend regular meetings, as well as to translate information and advice provided during the counseling and regular meetings to daily life, subsequently leading to a better compliance and greater health effects. In addition, employing new dietary counseling tools not requiring physical presence in a study center may also be an option for future studies. Furthermore, mechanisms underlying the elevated bacterial endotoxin levels found in the present study, as well as other studies, remain to be determined.

## Figures and Tables

**Table 1:** Characteristics of normal-weight healthy children and overweight children with and without non-alcoholic fatty liver disease (NAFLD).

	NW	OW	NAFLD
*n*	36	73	16
Sex (male/female)	20/16	33/40	6/10
Ethnicity (Caucasian/Asian)	27/9	54/19	8/8
Age (years)	7.3 ± 0.2	7.6 ± 0.1	7.8 ± 0.3
Weight (kg)	27 ± 1	36 ± 1	40 ± 2 *
Height (cm)	1.26 ± 0.01	1.29 ± 0.01	1.33 ± 0.02
BMI (kg/m^2^)	16.7 ± 0.2	21.2 ± 0.2	22.4 ± 0.5 *
BMI-SD score	0.43 ± 0.09	1.90 ± 0.05	2.13 ± 0.11 *
Waist circumference (cm)	59 ± 1	72 ± 1	77 ± 2 *
Leptin (ng/mL)	2.3 ± 0.3	12.2 ± 1.0	9.2 ± 1.4
Adiponectin (µg/mL)	11.4 ± 0.9	10.4 ± 0.5	11.9 ± 2.2
Leptin/Adiponectin	0.3 ± 0.03	1.46 ± 0.17	1.21 ± 0.26
ALT (U/L)	19 ± 1	23 ± 1	24 ± 2
AST (U/L)	33 ± 1	31 ± 1	35 ± 2 *
Systolic blood pressure (mmHg)	103 ± 1	108 ± 1	111 ± 3
Diastolic blood pressure (mmHg)	62 ± 1	67 ± 1	70 ± 2
Triglycerides (mg/dL)	57 ± 3	81 ± 4	75 ± 6
HDL cholesterol (mg/dL)	57 ± 1	52 ± 1	48 ± 3
LDL cholesterol (mg/dL)	100 ± 3	117 ± 2	105 ± 6 *
Total cholesterol (mg/dL)	170 ± 4	185 ± 3	169 ± 6 *
Uric Acid (mg/dL)	3.6 ± 0.1	4.3 ± 0.1	4.1 ± 0.2
Insulin (µIU/mL)	9 ± 0.4	12 ± 0.6	16 ± 2.3
Fasting glucose (mg/dL)	85 ± 1	87 ± 1	85 ± 2
HOMA-IR	1.9 ± 0.1	2.5 ± 0.1	2.9 ± 0.4
Physical activity (h/week)	15 ± 1	14 ± 1	15 ± 2
Sedentary activity (h/week)	16 ± 2	22 ± 1	25 ± 4

Data are shown as absolute numbers or means ± SEM, * *p* < 0.05 compared to overweight children, NW children were not included in the statistical analysis but are shown for comparison. BMI: body mass index; BMI-SD score: BMI standard deviation score; ALT: alanine aminotransferase, AST: aspartate aminotransferase; HDL: high-density lipoprotein; LDL: low-density lipoprotein; HOMA-IR: homeostatic model assessment for insulin resistance; NW: normal-weight healthy children; OW: overweight children without NAFLD; NAFLD: overweight children with NAFLD. Underreporters were excluded from the analysis.

**Table 2:** Nutritional intake of normal-weight healthy children and overweight children with and without NAFLD.

	NW	OW	NAFLD
*n*	36	73	16
Total energy intake (kcal/day)	1900 ± 70	1853 ± 47	2101 ± 105 *
Total fat intake (g/day)	78 ± 4	77 ± 3	86 ± 6
Total protein intake (g/day)	59 ± 3	63 ± 2	68 ± 4
Total CHO intake (g/day)	242 ± 10	227 ± 7	257 ± 16
Fructose (g/day) ^a^	55 ± 3	47 ± 2	61 ± 6 *
Glucose (g/day) ^b^	49 ± 2	42 ± 2	53 ± 6 *
Fiber intake (g/day)	15 ± 1	14 ± 1	18 ± 2

Data are shown as means ± standard error of the mean (SEM); * *p* < 0.05 compared to overweight children; NW children were not included in the statistical analysis, but are shown for comparison. CHO: carbohydrate; NW: normal-weight healthy children; OW: overweight children without NAFLD; NAFLD: overweight children with NAFLD, ^a^ free fructose and fructose derived from sucrose; ^b^ free glucose and glucose derived from sucrose. Underreporters were excluded from the analysis.

**Table 3:** Dietary pattern of normal-weight children and overweight children with and without NAFLD.

	NW	OW	NAFLD
Beverages (kcal/day)	161 ± 18	132 ± 10	208 ± 35 *
Fruits/dried fruits (kcal/day)	86 ± 12	80 ± 7	130 ± 30
Vegetables/legumes (kcal/day)	27 ± 5	30 ± 4	24 ± 5
Potatoes/pasta/rice (kcal/day)	210 ± 33	170 ± 14	201 ± 36
Bread (kcal/day)	259 ± 22	257 ± 18	262 ± 48
Spreads (kcal/day)	141 ± 23	103 ± 10	84 ± 18
Bakery goods (kcal/day)	206 ± 28	217 ± 27	167 ± 33
Cereals (kcal/day)	127 ± 35	113 ± 12	146 ± 5
Meat (kcal/day)	216 ± 24	205 ± 18	249 ± 46
Milk and dairy (kcal/day)	161 ± 16	144 ± 11	168 ± 37
Cheese and quark (kcal/day)	92 ± 15	104 ± 11	77 ± 12
Oils, margarines, and butter (kcal/day)	94 ± 12	103 ± 9	136 ± 15
Sweets and sugar (kcal/day)	154 ± 26	178 ± 15	145 ± 17
Desserts (kcal/day)	157 ± 38	111 ± 21	173 ± 80
Convenience food (kcal/day)	231 ± 28	324 ± 37	317 ± 63

Data are shown as means ± SEM; * *p* < 0.05 compared to overweight children without NAFLD; NW children were not included in the statistical analysis, but are shown for comparison. NW: normal-weight healthy children; OW: overweight children without NAFLD; NAFLD: overweight children with NAFLD.

**Table 4:** Anthropometric and metabolic parameters of overweight children with NAFLD before and after dietary intervention.

	Healthy Children	NAFLD Children			
	NW (*n* = 36)	Control (*n* = 6)		Intervention (*n* = 7)	
		Baseline	After 1 Year	Baseline	After 1 Year
Sex (male/female)	20/16	3/3		4/2	
Ethnicity (Caucasian/Asian)	27/9	2/4		5/1	
Age (years)	7.3 ± 0.2	8.0 ± 0.3	9.0 ± 0.5 *	7.5 ± 0.4	8.7 ± 0.4 *
BMI-SD score	0.43 ± 0.09	1.9 ± 0.1	1.6 ± 0.2 *	2.2 ± 0.2	2.0 ± 0.3
Waist circumference (cm)	59 ± 1	77 ± 4	77 ± 4	76 ± 3	83 ± 3 *
Leptin (ng/mL)	2.3 ± 0.3	9.5 ± 3.0	16.6 ± 4.8	8.8 ± 2.5	17.5 ± 5.7
Adiponectin (µg/mL)	11.4 ± 0.9	7.6 ± 1.4	6.9 ± 0.7	18.5 ± 4.3	8.3 ± 1.9
Leptin/adiponectin	0.3 ± 0.03	1.6 ± 0.6	2.5 ± 0.8	0.7 ± 0.3	2.0 ± 0.3 *
LBP (µg/mL)	23.6 ± 1.3	28.3 ± 4.0	30.2 ± 3.2	28.8 ± 4.5	25.6 ± 2.6
Active PAI-1 (U/L)	6.8 ± 0.8	18.2 ± 5.6	18.2 ± 8.6	17.0 ± 4.0	9.6 ± 1.4
ALT (U/L)	19 ± 1	26 ± 4	26 ± 3	20 ± 1	23 ± 3
AST (U/L)	33 ± 1	38 ± 5	30 ± 2	32 ± 3	33 ± 3
Systolic blood pressure (mmHg)	103 ± 1	111 ± 5	113 ± 5	107 ± 4	105 ± 5
Diastolic blood pressure (mmHg)	62 ± 1	69 ± 3	70 ± 6	69 ± 4	65 ± 4 *
Triglycerides (mg/dL)	57 ± 3	80 ± 7	85 ± 13	74 ± 15	89 ± 27
HDL cholesterol (mg/dL)	57 ± 1	48 ± 3	52 ± 4	56 ± 4	57 ± 5
LDL cholesterol (mg/dL)	100 ± 3	109 ± 8	115 ± 10	102 ± 13	116 ± 11 *
Total cholesterol (mg/dL)	160 ± 4	176 ± 7	174 ± 12	170 ± 12	187 ± 10 *
Insulin (µIU/mL)	9 ± 0.4	13 ± 1	15 ± 3	13 ± 4	16 ± 4
Fasting glucose (mg/dL)	85 ± 1	78 ± 4	86 ± 3	85 ± 2	86 ± 3
HOMA-IR	1.9 ± 0.1	2.5 ± 0.3	3.3 ± 0.8	2.8 ± 0.9	3.4 ± 0.9
Physical activity (h/week)	15 ± 1	17 ± 4	17 ± 5	14 ± 2	14 ± 2
Sedentary activity (h/week)	16 ± 2	23 ± 7	25 ± 4	24 ± 4	18 ± 4

Data are shown as absolute numbers or means ± SEM; * *p* < 0.05 compared to the respective baseline value. BMI-SD score: BMI standard deviation score; LBP: lipopolysaccharide binding protein; PAI-1: plasminogen activator inhibitor-1; ALT: alanine aminotransferase; AST: aspartate aminotransferase; HDL: high-density lipoprotein; LDL: low-density lipoprotein; HOMA-IR: homeostatic model assessment for insulin resistance. NW children were not included in the statistical analysis, but are shown for comparison. Underreporters were excluded from the analysis.

## Supplementary Materials

The following are available online at http://www.mdpi.com/2072-6643/10/9/1329/s1, Table S1: Nutritional intake of overweight children with NAFLD enrolled in the feasibility study at baseline.
