# Supplementary material for: Non-Alcoholic Fatty Liver Disease in Overweight Children: Role of Fructose Intake and Dietary Pattern
Source: Nutrients. 2018 Sep 19;10(9):1329. doi: 10.3390/nu10091329 (PMC6165138; doi:10.3390/nu10091329)
Supplement: Supplementary file 1 [file nutrients-10-01329-s001.pdf]

Supplemental Table S1. Nutritional intake of overweight children with NAFLD enrolled in the feasibility study at baseline.

	NW	Control	Intervention
n	36	6	6
**Total energy intake (kcal/d)**	1900 ± 70	2125 ± 203	2166 ± 121
**Total fat intake (g/d)**	78 ± 4	91 ± 10	87 ± 4
**Total protein intake (g/d)**	59 ± 3	64 ± 6	31 ± 11
**Total CHO intake (g/d)**	242 ± 10	255 ± 30	266 ± 24
**Total fructose (g/d)**[±]	55 ± 3	69 ± 13	54 ± 5
**Total glucose (g/d)**[$]	49 ± 2	62 ± 12	48 ± 5
**Fiber intake (g/d)**	15 ± 1	22 ± 3	18 ± 3

Data are shown as absolute numbers or means ± SEM, NW: normal weight children. NW children were not included in the statistical analysis but are shown for comparison. [±]free fructose and fructose deriving from sucrose, [$]free glucose and glucose deriving from sucrose. `Under-reporters´ were excluded from the analysis. Data of ´under-reporters´ and children that dropped out of the study were excluded from the analysis.
